# Supplementary material for: Landscape of histone modifications in a sponge reveals the origin of animal cis-regulatory complexity
Source: eLife. 2017 Apr 11;6:e22194. doi: 10.7554/eLife.22194 (PMC5429095; doi:10.7554/eLife.22194)
Supplement: Supplementary File 1. — DOI: http://dx.doi.org/10.7554/eLife.22194.043 [file elife-22194-supp1.docx]

**Sequences of the primers used for ChIP-quantitative PCRs (ChIP-qPCRs)**

*H3K4me1*

Primer 1 (Fw^a^) 5’ AGTGGCCATTGCAATTAGTG

Primer 1 (Rev^b^) 5’ TTCTAAGCTCAAACCCACTGC

Primer 2 (Fw) 5’ TGTGGCTGTGATGTAGTAGTTCTG

Primer 2 (Rev) 5’ AATTGGCACATTCCAAGGAC

Primer 3 (Fw) 5’ TTGCAACACTATAAGTGAAACCTTG

Primer 3 (Rev) 5’ GGCATATTTATGGTGGCTCATAG

Primer 4 (Fw) 5’ AGCAAGCTCTGTGTGTGTGAG

Primer 4 (Rev) 5’ GCCACTACCAGCACACTCC

Primer 5 (Fw) 5’ GGGAACTAGGAGCAGTACCAGTAG

Primer 5 (Rev) 5’ TGGCACCCATTCACTATCAG

Primer 6 (Fw) 5’ CGCAGTATTGCTGGAATGTG

Primer 6 (Rev) 5’ ACCTCAAAGCCATTCGTCTG

Primer 7 (Fw) 5’ ATTTGCTGGAGAATGGTGCT

Primer 7 (Rev) 5’ GCTTTCAGGAGACCTAATGAGTAA

Primer 8 (Fw) 5’ GGCAGTGCCTGTGTTAGGAT

Primer 8 (Rev) 5’ CCCAATACACACATTCCCAAT

Primer 9 (Fw) 5’ TTCTCCCACAACTACCACCA

Primer 9 (Rev) 5’ TGTCTTGTGATTACAGCCAAATG

Primer 10 (Fw) 5’ TTTGGTTTGCCTTACAAGGTG

Primer 10 (Rev) 5’ TTGCCTGAAGGCACTAACTG

Primer 11 (Fw) 5’ TGACCAAATGTTGTCATCATCA

Primer 11 (Rev) 5’ TTTGCATATCTCGCAACTTT

Primer 12 (Fw) 5’ GGATAGCAGCTGGAGACGAC

Primer 12 (Rev) 5’ AGCATGGATTCCTTTGCACT

Primer 13 (Fw) 5’ CCACCACAGGTCATTAGGTATC

Primer 13 (Rev) 5’ AGGTGTGTTGAGCCTGACCT

Primer 14 (Fw) 5’ ATCCTTTCCTGCAGTGTTGG

Primer 14 (Rev) 5’ GGTAGCTCACAGCATGTGATTG

Primer 15 (Fw) 5’ TAGGTGTGCAGTGCTGGTTC

Primer 15 (Rev) 5’ CCTCAATGCTCTTCCTCTGTG

Primer 16 (Fw) 5’ GAGACGGTAACTGGGAGCAG

Primer 16 (Rev) 5’ CCTTACTGCTCCGTCAAAGG

Primer 17 (Fw) 5’ CACCGTCAATCTCACCTGAA

Primer 17 (Rev) 5’ TATCGTATGAGGCGGCTTTC

Primer 18 (Fw) 5’ CCTGACAGTGATTCGTACATCC

Primer 18 (Rev) 5’ AGGAACATTGGTCAGTGTGATG

Primer 19 (Fw) 5’ TAGCATGCAACTCCAGCATC

Primer 19 (Rev) 5’ CCATAGTGTCCTTCCCAAAGC

Primer 20 (Fw) 5’ GGAGTTGGTGGAAGTGGAAA

Primer 20 (Rev) 5’ AGGAATATGGTGCCCACTGA

Primer 21 (Fw) 5’ AGAGGAGACGGCAGCTATGA

Primer 21 (Rev) 5’ CAATATGAAGCACATCCCAAA

*H3K27ac*

Primer 1 (Fw) 5’ AATCCGCCACAGTAACCATC

Primer 1 (Rev) 5’ CGTATAGAGGTCACCGGATATTG

Primer 2 (Fw) 5’ ACAGCCACCGGAAAATAACA

Primer 2 (Rev) 5’ TCATAATCAGGATACTAACAGCTCA

Primer 3 (Fw) 5’ AATCGCTCAAGATTGTCACG

Primer 3 (Rev) 5’ AAGAACAAGGTTGCAGCAGAG

Primer 4 (Fw) 5’ TATGAGCTAATGGGGCCAAC

Primer 4 (Rev) 5’ GCGGGTGGACAAATAAAGC

Primer 5 (Fw) 5’ AAAGTGCAATGACAACCGAAC

Primer 5 (Rev) 5’ AGCAGTGCCAGAGTGACAAG

Primer 6 (Fw) 5’ CGCAGTATTGCTGGAATGTG

Primer 6 (Rev) 5’ ACCTCAAAGCCATTCGTCTG

Primer 7 (Fw) 5’ CTATGGGATGGACAGGGATG

Primer 7 (Rev) 5’ AGGAGTGTCTTCATTACCTGGAG

Primer 8 (Fw) 5’ CCCTAGAGCAGAGTCCATTCC

Primer 8 (Rev) 5’ CACTACTGGCCCTCTCCAAC

Primer 9 (Fw) 5’ GATTATTTGGGTGGGTGCAG

Primer 9 (Rev) 5’ GCAAATGAGGTTGAATTATGTCTT

Primer 10 (Fw) 5’ AGCAGGTACCCCTACACAGC

Primer 10 (Rev) 5’ GAGCCGGATAGACGACTCTG

Primer 11 (Fw) 5’ GCATGTAGCTATTGCCATCC

Primer 11 (Rev) 5’ TTGCTGGTTTTCTTAGTGCTTG

Primer 12 (Fw) 5’ CTCTCCTGGCAAAAGACACC

Primer 12 (Rev) 5’ TAGGGGTGAGGAGAGAGTCG

Primer 13 (Fw) 5’ TGCCACAGTCACCCAATATG

Primer 13 (Rev) 5’ ATGTGGGCGAGTGGGTATAG

Primer 14 (Fw) 5’ CAATCCATGTCCCTTGTGTG

Primer 14 (Rev) 5’ AAAGCGGCAGTAGCTTTGAG

Primer 15 (Fw) 5’ TCCTCCAATACCAGCGAATC

Primer 15 (Rev) 5’ CCACACACTCCAACCATGTC

Primer 16 (Fw) 5’ TCATGTGCCACTCAAAGCTC

Primer 16 (Rev) 5’ TGTGCAGGCTCATACTTAGGG

Primer 17 (Fw) 5’ ACCTGGGATCAGCTTCAGTG

Primer 17 (Rev) 5’ GAAAGGCTTGCTTTCCTGTG

Primer 18 (Fw) 5’ CCCCAATTGATGTCTTTTCC

Primer 18 (Rev) 5’ AAGGATGGGGCCACATTTAC

Primer 19 (Fw) 5’ AAAGCATTGTCGGCAGTAGG

Primer 19 (Rev) 5’ AAGGTGACCTGTCCCCAAC

Primer 20 (Fw) 5’ GTCCTTAGTCCCACCCATTG

Primer 20 (Rev) 5’ TGGAGTAGGACAGGCTCCTC

*H3K4me3*

Primer 1 (Fw) 5’ CACGGGTTCTGGTATTGTTG

Primer 1 (Rev) 5’ CCTCCCCTTTGCTTTAATGTC

Primer 2 (Fw) 5’ CTCCCTCTTACCGTCTGCTC

Primer 2 (Rev) 5’ TTATGTCGTTCCGGCTGTG

Primer 3 (Fw) 5’ GAAGCTTTCCCTTCCTCTCC

Primer 3 (Rev) 5’ TAGGCAGGTATCCGTTGGTC

Primer 4 (Fw) 5’ TGCCGCATAGATTCTCAGTG

Primer 4 (Rev) 5’ GCAAGCTACTTGGGCATCAG

Primer 5 (Fw) 5’ TCGTTGCTCTCAGCCATTC

Primer 5 (Rev) 5’ ACTCCTAGTGGTCGGGACAG

Primer 6 (Fw) 5’ AAAGCTGCCACACGTAGTCC

Primer 6 (Rev) 5’ GACTTTAGCTGACACTCCTAGTGG

Primer 7 (Fw) 5’ GGTTAACGAGGCGAGCTATG

Primer 7 (Rev) 5’ GAGGACTGACGATGCAGTTG

Primer 8 (Fw) 5’ ATGGAGGACAAACGCAGTTC

Primer 8 (Rev) 5’ TCATTGTAGCGTGCCTCCTC

Primer 9 (Fw) 5’ CGTCATCAGAGAGTGCATCAG

Primer 9 (Rev) 5’ GCAAGAGAAAGGCTGCTTATTG

Primer 10 (Fw) 5’ CCATCAGTACTGGCATCACG

Primer 10 (Rev) 5’ TGTACGTCCCTTGCTTCTCC

*H3K27me3*

Primer 1 (Fw) 5’ TGCAGTCAACTGGATGTATGG

Primer 1 (Rev) 5’ TTGGGTTCAAAGCTCAAAGG

Primer 2 (Fw) 5’ AACAGACTCCCGCAGTTCTC

Primer 2 (Rev) 5’ GCAGTAGGAGTGGACCGAAG

Primer 3 (Fw) 5’ TGGCTGTGTGTTGACGCTAC

Primer 3 (Rev) 5’ CAACCGTGTCCTACCTCCTC

Primer 4 (Fw) 5’ ATCAAAGGTCAACCGTGTCC

Primer 4 (Rev) 5’ CGGTCAGGTCGGTCAGTAAA

Primer 5 (Fw) 5’ TGAAGCCAAGACATGAAGGA

Primer 5 (Rev) 5’ TTTGGGGCTTTGTGTATGTG

Primer 6 (Fw) 5’ AGCAGAGAGAACGACGGAAG

Primer 6 (Rev) 5’ TGCTAAGCGCTCCATTTCTT

Primer 7 (Fw) 5’ AAGGCGACAAAGGCTGTAAA

Primer 7 (Rev) 5’ CGCCATTTTCTCACTCCAAT

Primer 8 (Fw) 5’ CACTGTCCCACATCACATCC

Primer 8 (Rev) 5’ CCAGATGTAACAGGCAACCA

Primer 9 (Fw) 5’ AGTTGAGGAAACGGCCAAG

Primer 9 (Rev) 5’ GGCGACGTCTTCTGCTAGTT

Primer 10 (Fw) 5’ TTTGGATTTCTCAAGCATCG

Primer 10 (Rev) 5’ TGAACGTAGCTTCAACAGCAG

*Intergenic regions*

Primer 1 (Fw) 5’ CAATGCACAGCACTATTTATCAGTT

Primer 1 (Rev) 5’ CATGCAACCGTGTGATTACC

Primer 2 (Fw) 5’ GATTTCTTCATATAGTGGCAATGG

Primer 2 (Rev) 5’ CCAAATTTAGTGGTCGCTTCTC

^a^Forward primer.

^b^Reverse primer.
